# Pharmacotherapy of high-risk population for developing psychosis

**DOI:** 10.1192/j.eurpsy.2023.113

**Published:** 2023-07-19

**Authors:** P. Mohr

**Affiliations:** ^1^National Institute of Mental Health, Czechia, Klecany; ^2^Third Faculty of Medicine, Charles University, Prague, Czech Republic

## Abstract

**Abstract:**

Early interventions in high-risk population for psychotic disorder target both conversion rates and functional impairments. Existing guidelines (European Psychiatric Association, NICE, Canadian) do not consider drug treatment as the first-line choice, pharmaceuticals mostly complement least restrictive, non-pharmacological approaches (e.g., CBT). Pharmacotherapy can address existing specific symptoms (mood fluctuations, anxiety, subclinical brief or attenuated psychotic symptoms); it is reserved mainly for individuals with more severe symptoms, those that do not respond to psychological treatments or are escalating. There are only a few randomized controlled trials with antipsychotics (olanzapine, risperidone, aripiprazole, ziprasidone, amisulpride), either as a monotherapy or in combination with other interventions. The results did not show a superiority of drug therapy in prevention of transition to psychosis over alternative strategies; long-term antipsychotic treatment with a primarily preventive aim is not generally recommended. Other pharmacological interventions also include experimental drugs or food supplements (omega-3 polyunsaturated fatty acids, cannabidiol, D-serine).

**Disclosure of Interest:**

None Declared

